# Outcome analysis of conservative treatment of a distal radius fracture with OPTIVOhand orthosis versus plaster cast: a randomized controlled trial

**DOI:** 10.1186/s12891-026-09585-4

**Published:** 2026-02-11

**Authors:** Maximilian Friederich, Julia Brunner, Elke Kirsch, Fabian Stuby, Alexander Zimmermann, Tina Histing, Philipp Hemmann

**Affiliations:** 1 Department of Traumatology and Reconstructive Surgery, BG Trauma Center Tuebingen, Eberhard Karls University Tuebingen, Schnarrenbergstr. 95, 72076 Tuebingen, Germany; 2 Faculty of Medicine, Eberhard Karls University Tuebingen, Geissweg 5, 72076 Tuebingen, Germany

**Keywords:** Distal radius fracture, orthosis, conservative fracture treatment, Immobilization, Patient satisfaction, Disabilities of arm shoulder and hand (DASH)

## Abstract

**Background:**

Distal radius fractures (DRF) are often immobilized using a conventional plaster cast although orthoses offer a time- and resource-saving alternative. This prospective randomized study compared the conservative DRF treatment using an orthosis (OPTIVOhand^®^) with plaster cast immobilization. Besides the maintenance of reduction result (primary endpoint), functional and subjective outcomes (secondary endpoints) were examined.

**Methods/ Design:**

53 patients with isolated DRF were randomized to the orthosis group (OG) or the control group (CG). The follow-up examinations included radiological, clinical, and functional evaluations (ROM, grip strength, DASH score, SF-36) as well as patient satisfaction questionnaire.

**Results:**

41 of the 53 patients included (OG: *n* = 21, CG: *n* = 20) were followed up until the 12 months follow-up. The rate of secondary dislocations was comparable in both groups (OG: *n* = 3; CG: *n* = 2; *p* > 0.05). Additionally, the OG showed significantly (*p* < 0.05) better subjective function (DASH score) 6 weeks and 3 months after injury, and a higher quality of life (SF-36 physical component summary) at 2 and 6 week follow-up. Patient satisfaction was significantly higher in OG and mean application time was significantly shorter (OG: 02:35 min vs. CG: 07:35 min; *p* < 0.001).

**Conclusion:**

This study’s functional and radiological results on conservative DRF treatment did not reveal a significant difference in maintenance of reduction result between modern orthoses and conventional plaster casts, while achieving higher patient satisfaction. Hence, orthoses offer a good alternative to plaster casts, especially for stable fracture types.

**Trial registration:**

German Clinical Trials Register, Identifier: DRKS00017695. Trial registration date 04.11.2019, (https://drks.de/search/en/trial/DRKS00017695)

**Supplementary Information:**

The online version contains supplementary material available at 10.1186/s12891-026-09585-4.

## Introduction

Distal radius fractures (DRF) are one of the most common fractures in the Western world, with an incidence of 200 per 100,000 people per year [1–3]. DRF have a bimodal age distribution [1] as they affect both younger men and older women after menopause [3–5]. In younger patients, fractures typically occur as a result of high-impact trauma, while osteoporosis-related fractures predominate in the older population [6]. Due to demographic change, a significant increase in incidence is expected in the coming years [6–8]. With a future increase in the number of geriatric patients, conservative treatment will become increasingly important. It has already been shown in older patients that fractures healed after 6 weeks following both conservative and surgical treatment. Moreover, the functional results in the Disabilities of the Arm, Shoulder and Hand score (DASH) and Patient Rating Wrist Evaluation were satisfactory in conservatively treated versus surgically treated patients [9].

In literature, complication rates for surgical DRF treatment between 5% and 35% were reported [10, 11]. In this context, the most common complications are nerve irritation (up to 17% [12]), complex regional pain syndrome (CRPS) (< 3% [12, 13]), flexor tendon irritation (0%-12% [14]), extensor tendon irritation (up to 1% [13]), delayed healing and pseudarthrosis (< 0.2% [15]), and malalignment after healing (up to 17% [16]). In contrast, the most common complication of conservative treatment is CRPS [17]. Other complications may include skin ulcers due to incorrect cast modelling, ruptures of the extensor pollicis longus tendon (< 1%), or misclassification with subsequent secondary dislocation [17]. Quadlbauer et al. showed in 2017 that earlier mobilization after surgical treatment ultimately led to better functional results [18].

In their study, Stuby et al. evaluated the effects of a dynamic, vacuum-assisted orthosis compared to a classic dorsal plaster cast on the postoperative course after palmar plate osteosynthesis. They showed that using an orthosis offers advantages in terms of early range of motion and patient satisfaction without increasing the risk of complications [19]. Al Khudairy et al. also confirmed in their study that orthosis use in stable distal radius fractures leads to good radiological results without dislocation of the fracture site. As in the study by Stuby et al., their work also highlighted the high level of patient satisfaction, as well as other everyday activities (e.g., showering) that could be performed easily with orthoses [19, 20]. However, there are also studies with 3D-printed orthoses that had to be discontinued early because they showed a large number of secondary dislocations [21]. Thus, DRF treatment with orthoses is still considered critically in everyday clinical practice. Instead, classic immobilization using a plaster cast is primarily favoured.

The objective of this randomized controlled trial with parallel group design was to evaluate the conservative treatment of DRF using orthoses compared to conventional plaster cast immobilization. The primary endpoint was the maintenance of reduction result, assessed radiographically. Secondary endpoints included functional outcome, patient satisfaction, and health-related quality of life.

## Materials and methods

The study was conducted in accordance with the Declaration of Helsinki and approved by the local ethics committee (Project no. 339/2019BO2, 17.10.2019). It was registered in the German Clinical Trials Registry (DRKS00017695, https://drks.de/search/en/trial/DRKS00017695, registration date 04.11.2019). In this prospective, randomized, monocentric study with parallel group design a total of 53 subjects were enrolled in the Department of Traumatology and Reconstructive Surgery at a BG Trauma Center (Germany) between August 2020 and March 2023. Informed consent was obtained from all individual participants included in the study. The number of cases was determined a priori by a power analysis (*N* = 50). The inclusion and exclusion criteria are summarized in Table 1. This study is reported according to the Consolidated Standards of Reporting Trials (CONSORT) guidelines [22] (see Supplement-1).

**Table 1:** Inclusion and exclusion criteria

Inclusion Criteria	• Distal radius fractures AO 23 A2- C3 • No soft tissue damage according to Anderson and Gustilo (Gustilo I) • Patient is able to actively support conservative treatment • Conservative treatment feasible
Exclusion Criteria	• Surgical intervention required • Wrist deformity • Sensory loss or reduced sensation of the hand • Neurodegenerative diseases including dementia • Tumor diseases/pathological fractures • Additional fractures and concomitant injuries • Age < 18 years or > 80 years • Severe soft tissue injuries (Gustilo Grade II and III) • Lack of compliance or inability to support conservative therapy

**Fig. 1:**
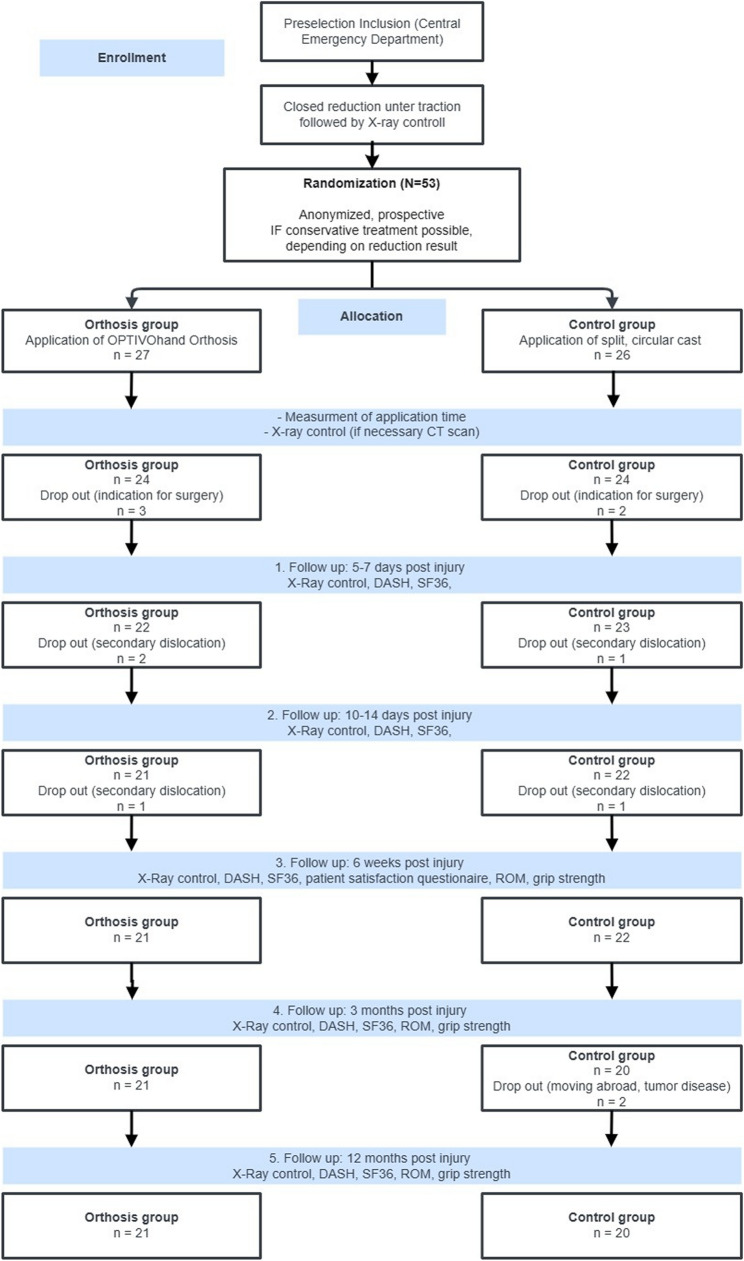
Patient flowchart according to CONSORT guidelines to represent the study design and patient flow including justified drop out

Randomization was performed using a computer-assisted random number generator. Before starting the study, 55 participation certificates were labelled, folded, and placed in consecutively numbered envelopes. These certificates were sealed by an independent study nurse. After informing the patients and obtaining their written consent, the attending physician opened the next envelope in numerical order in the emergency room. At this point, the physician had no knowledge of the assigned type of treatment. In this way, the patients were randomly assigned to the orthosis group (OG) or the control group (CG). Until the final treatment procedure, neither the staff nor the patient knew the type of procedure (cast or orthosis). Due to the type of immobilization, blinding of patient and physician was not possible during the following treatment and examinations. The study participants received an individual identification number, and all data were pseudonymized. For a better overview, a patient flowchart was created for the study protocol (Fig. 1).

The fracture was repositioned in the hanging, if necessary, and then treated with either a split forearm cast or an orthosis. Both were worn for 6 weeks. Patients with a split forearm cast were switched to a closed hard cast (Cellacast Xtra, Lohmann & Rauscher International, Rengsdorf, Germany) after 5–7 days. The orthosis used, OPTIVOhand^®^ (OPED GmbH, Valley, Germany), is made of textile fibres (polyester), plastic reinforcements, metal splints (aluminium), and padding foam. It can be used for both the left and right wrists. The interchangeable aluminium splint allows the appropriate side to be adjusted. The application procedure of OPTIVOhand can be seen in Klopfer et al. [23]. The time required to apply the orthosis or forearm cast was measured during initial treatment. The hardening and splitting of the cast were not considered in the time measurement. After immobilization, the clinic’s standard operating procedure required an X-ray, or a CT scan if necessary, to rule out secondary dislocation due to the application of cast or orthosis. If the need for surgical treatment was determined, the patient was excluded from further participation in the study.

**Fig. 2:**
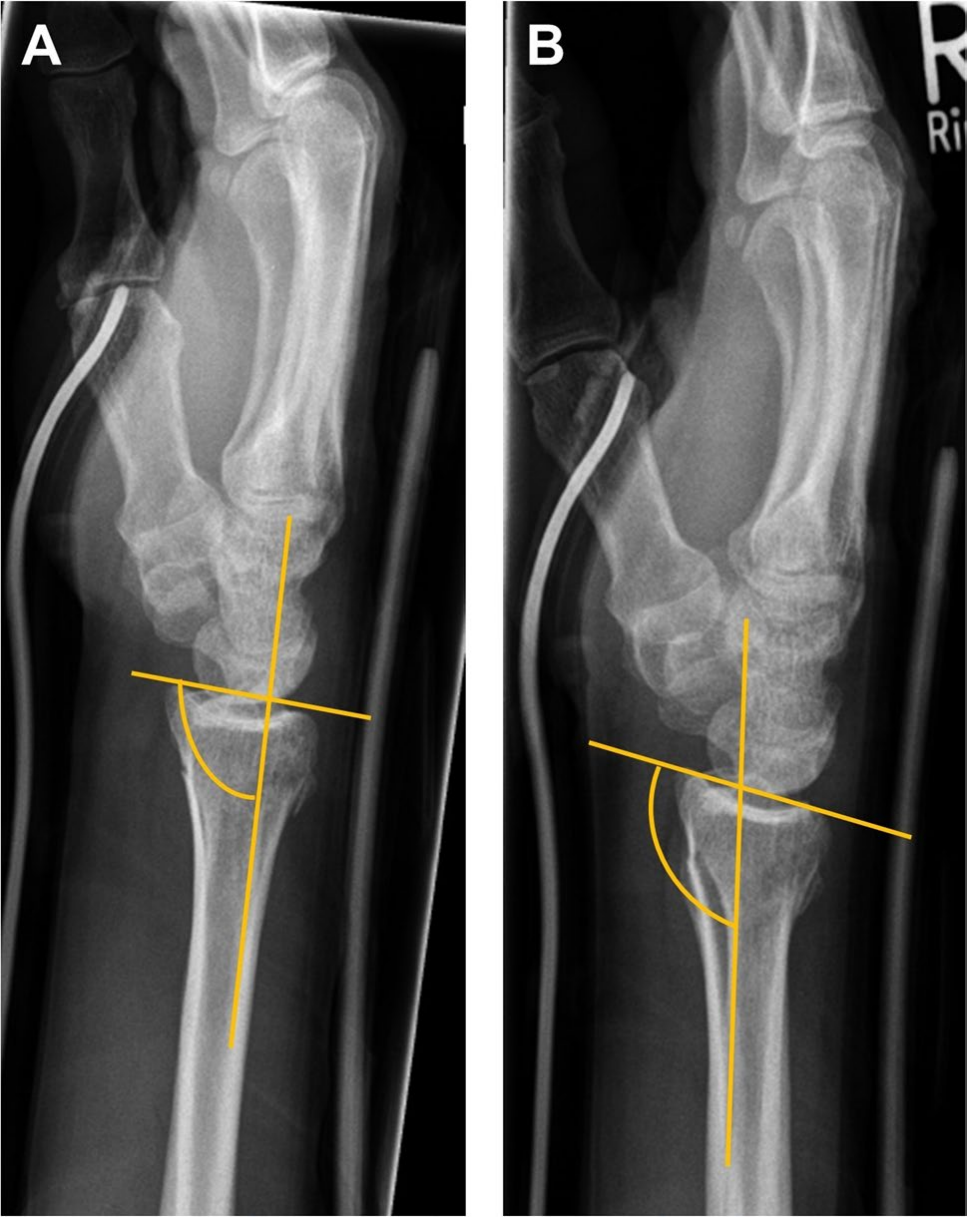
Reduction results after orthosis application (**A**) and secondary dislocation of the same patient at second follow-up (10–14 day after injury) indicated by an increase in Böhler-II-angle > 10° (**B**)

Follow-up examinations were conducted 5 to 7 days, 10 to 14 days, 6 weeks, 3 months, and 1 year after the intervention. The primary endpoint was defined as the maintenance of the reduction result. Thus, radiological monitoring was performed using X-rays in two planes of the affected wrist in each follow-up examination. The presence of a secondary dislocation was defined using the Böhler-II-angle with a loss of the reduction results by more than 10° palmar inclination (Fig. 2). The loss of reduction result within 14 days after injury required surgical treatment with palmar plate osteosynthesis.

Secondary outcome parameters included the evaluation of health-related quality of life using the Short Form 36 questionnaire (SF36, German version) [24] and the assessment of functional limitations using the Disability of the Arm, Shoulder, and Hand questionnaire (DASH, German version) [25]. Two weeks after injury and after removal of the orthosis and forearm cast in the sixth week, all patients received a questionnaire regarding patient satisfaction that included different aspects such as pain progression, handling, hygiene, fit, pressure discomfort, and aesthetics, which were rated using a 5-point Likert scale, in which better rating is represented with increasing numbers. This questionnaire was not validated; however, it was previously used in the studies of Stuby et al. [19] and Klopfer et al. [23] (see Supplement-2). Additionally, wrist range of motion (ROM) and grip strength were measured in the remaining follow-up examinations after the immobilization device was removed. Any other adverse events or patient harm were recorded on an ad hoc basis.

The statistical analysis was performed using Microsoft Excel, version 16.94 (Microsoft Corporation, Redmond, WA, USA) and IBM SPSS Statistics, version 29.0.2.0 (IBM Corporation, Armonk, New York, NY, USA). The primary endpoint – maintenance of the reduction result – was analysed by the Χ²-test and Fischer’s exact test for the two categorical parameters group allocation and secondary dislocation. In addition, Cramér’s V was calculated as a measure of effect size to estimate the strength of such correlations. The analysis of secondary endpoints followed a per-protocol approach including only complete cases since a secondary dislocation and the following indication for surgery would be contrary to the aimed conservative treatment. The data of secondary endpoints were tested for normal distribution using the Shapiro-Wilk test and additionally for the equality of variances using the Levene test. If both requirements were met, independent t-test were used for mean comparison. If requirements were not met, the Mann-Whitney U test was used for group comparison. In one case, the Monte Carlo method was applied for significance estimation due to a very small and unevenly distributed sample.Unless otherwise stated, values are presented as mean ± SD. The significance level was set uniformly at *p* < 0.05.

## Results

A total of 53 patients with isolated DRF were included in the study between August 2020 and March 2023. 26 patients were assigned to the CG while 27 patients were assigned to the OG. There was no change between the immobilization types during treatment. Patient characteristics and fracture classification are described in Table 2. There was a significant difference in age between the OG and CG (Mann-Whitney U-test, *p* = 0.025) where patients in the CG were older on average than the patients in the OG. In the CG the left side was more frequently affected (61.5%) whereas in the OG it was the right side (59.3%). This difference was not significant.

**Table 2:** Patient characteristics and fracture classification

Patients (f/m)		plaster	orthosis	*p*
		26 (18 / 8)	27 (15 / 12)	0.305
Age [years]		61 ± 15	50 ± 19	0.025
Drop out (f/m)		6 (5 / 1)	6 (6 / 0)	1.000
Injured side (l/r)		16 / 10	11 /16	0.130
Type of fracture – AO 23		n	n	
A	1	1	0	0.617
	2	9	10	
	3	4	3	
B	1	1	3	0.673
	2	4	5	
	3	1	3	
C	1	4	1	1.000
	2	1	1	
	3	1	1	

The fracture types were classified according to the AO classification. Type A fractures were the most common ones (51%, *n* = 27; CG *n* = 14, OG *n* = 13). There was no significant difference between the fracture severity according to the AO classification for type A (*p* = 0.617), type B (*p* = 0.673), and type C injuries (*p* = 1.000).

The primary endpoint of the study was the maintenance of the reduction result under conservative therapy with orthosis and plaster cast. Overall, there were only isolated cases of secondary dislocations in both groups (CG: *n* = 2; OG: *n* = 3) but no significant relationship was found between the type of immobilization and the number of secondary dislocations (*p* > 0.05). Complete follow-up data of secondary endpoints were collected from 21 patients in the OG and 20 patients in the CG.

### Study-specific questionnaire

There was no significant difference in pain progression between the groups. However, a significant difference was observed in the parameter wrist performance and functionality after 10–14 days (*p* = 0.012), 6 weeks (*p* < 0.001), and 3 months (*p* = 0.009) after injury in favour of the orthosis treatment. Further, the OG showed a significant higher overall physical resilience during the follow-up examinations after 6 weeks and 3 months (*p* = 0.04). Regarding patient satisfaction, the orthosis also showed a significant advantage over the cast in terms of personal hygiene (5–7 days/ 10–14 days after injury: *p* < 0.001), adaptability of the treatment (5–7 days after injury: *p* = 0.013, 10–14 days after injury: *p* < 0.001) and dressing (5–7 days after injury: *p* = 0.001, 10–14 days after injury: *p* < 0.002) in the early phase of the conservative treatment. Simultaneously, no significant difference between the OG and CG was found regarding pressure discomfort and appearance (aesthetics). Mean results of the study-specific questionnaire are presented in Table 3.

**Table 3:** Mean data of clinical scores, study-specific questionaire and clinical funtion after 5–7 days, 10–14 days, 6 weeks, 3 months and 12 months

n (CG) = 20 n(OG) = 21			Time after Injury				
			5–7 days	10–14 days	6 weeks	3 months	12 months
Clinical Scores	SF36-MCS	CG	30.8 ± 6.3	32.3 ± 4.0	33.7 ± 4.7	30.1 ± 3.5	29.8 ± 5.3
		OG	28.8 ± 5.0	31.6 ± 5.6	31.5 ± 8.1	28.1 ± 5.1	28.9 ± 6.1
	SF36-PCS	CG	67.9 ± 6.2	**57.6 ± 7.3**	**46.4 ± 7.2**	55.2 ± 7.0	58.5 ± 5.5
		OG	59.7 ± 5.7	48.1 ± 9.1 *	50.8 ± 8.9 *	59.2 ± 4.5	59.7 ± 6.0
	DASH	CG	1.6 ± 3.9	70.2 ± 7.2	**61.8 ± 18.5**	**13.3 ± 16.6**	2.7 ± 5.5
		OG	3.7 ± 10.5	62.1 ± 19.8	46.5 ± 26.0 *	4.2 ± 5.2 *	2.7 ± 9.7
Study-specific Questionaire	performance & functionality	CG		**1.5 ± 0.8**	**1.6 ± 0.9**	**3.6 ± 1.4**	4.5 ± 1.2
		OG		2.4 ± 1.5 *	2.8 ± 1.2 ***	4.6 ± 1.0 ***	4.9 ± 0.5
	physical resilience	CG		3.1 ± 1.4	**3.8 ± 1.5**	**4.2 ± 1.5**	4.9 ± 0.5
		OG		3.2 ± 1.1	3.9 ± 1.5 *	4.8 ± 0.6 *	4.9 ± 0.5
	personal hygiene	CG		**2.2 ± 1.3**	**1.9 ± 1.2**		
		OG		3.1 ± 1.1 ***	3.5 ± 1.3 ***		
	adaptability	CG		**3.6 ± 1.4**	**3.9 ± 1.3**		
		OG		2.0 ± 1.1 ***	1.7 ± 1.3 ***		
	dressing	CG		**2.2 ± 1.6**	**2.6 ± 1.3**		
		OG		4.1 ± 1.2 ***	4.0 ± 1.4 **		
	pressure discomfort	CG		3.4 ± 1.5	3.4 ± 1.5		
		OG		4.1 ± 1.1	3.4 ± 1.3		
Clinical Function	Grip Strength [kg]	CG			**7.1 ± 7.3**	21.1 ± 13.3	**25.1 ± 11.2**
		OG			20.8 ± 14.5***	29.6 ± 16.2	37.0 ± 15.8**
	ROM - Dorsal Extension [°]	CG			39.7 ± 14.7	60.5 ± 11.3	65.0 ± 14.7
		OG			44.8 ± 14.1	63.3 ± 11.3	66.5 ± 10.0
	ROM - Palmar Flexion [°]	CG			32.6 ± 11.9	49.8 ± 15.5	52.8 ± 10.5
		OG			44.5 ± 13.2	54.8 ± 11.3	64.0 ± 12.0
	ROM - Radial Abduction [°]	CG			17.9 ± 9.5	24.3 ± 6.5	27.8 ± 8.8
		OG			19.0 ± 7.0	26.8 ± 7.5	27.3 ± 8.0
	ROM - Ulnar Abduction [°]	CG			28.4 ± 11.6	38.0 ± 10.1	42.8 ± 11.7
		OG			33.0 ± 10.9	39.5 ± 10.2	41.3 ± 7.9

Control Group (CG); Orthosis Group (OG); Short Form 36 - Mental Component Score (SF36-MCS); Short Form 36 - Physical Component Score (SF36-PCS); Disabilities of Arm, Shoulder and Hand Score (DASH); Range of Motion (ROM). All values are presented as mean ± SD. * denotes significant difference between both groups: * *p* < 0.05, ** *p* < 0.01, ***, *p* < 0.001

### Quality of life SF-36

The two summary scores PCS (physical health component summary score) and MCS (mental health component summary score) were used for SF-36 evaluation. They summarize various items of the SF-36 questionnaire. Looking at the PCS score at first and last follow-up after injury, no significant difference was found (5–7 days after injury: *p* = 0.129, 12 months after injury: *p* = 0.133). However, we observed a significant difference in favour of the orthosis when evaluating the examinations after 10–14 days and 6 weeks after injury (*p* = 0.032). For the MCS score, no significant differences were found between OG or the CG at any time point. Mean results of SF-36 subscales are presented in Table 3.

### DASH

**Fig. 3:**
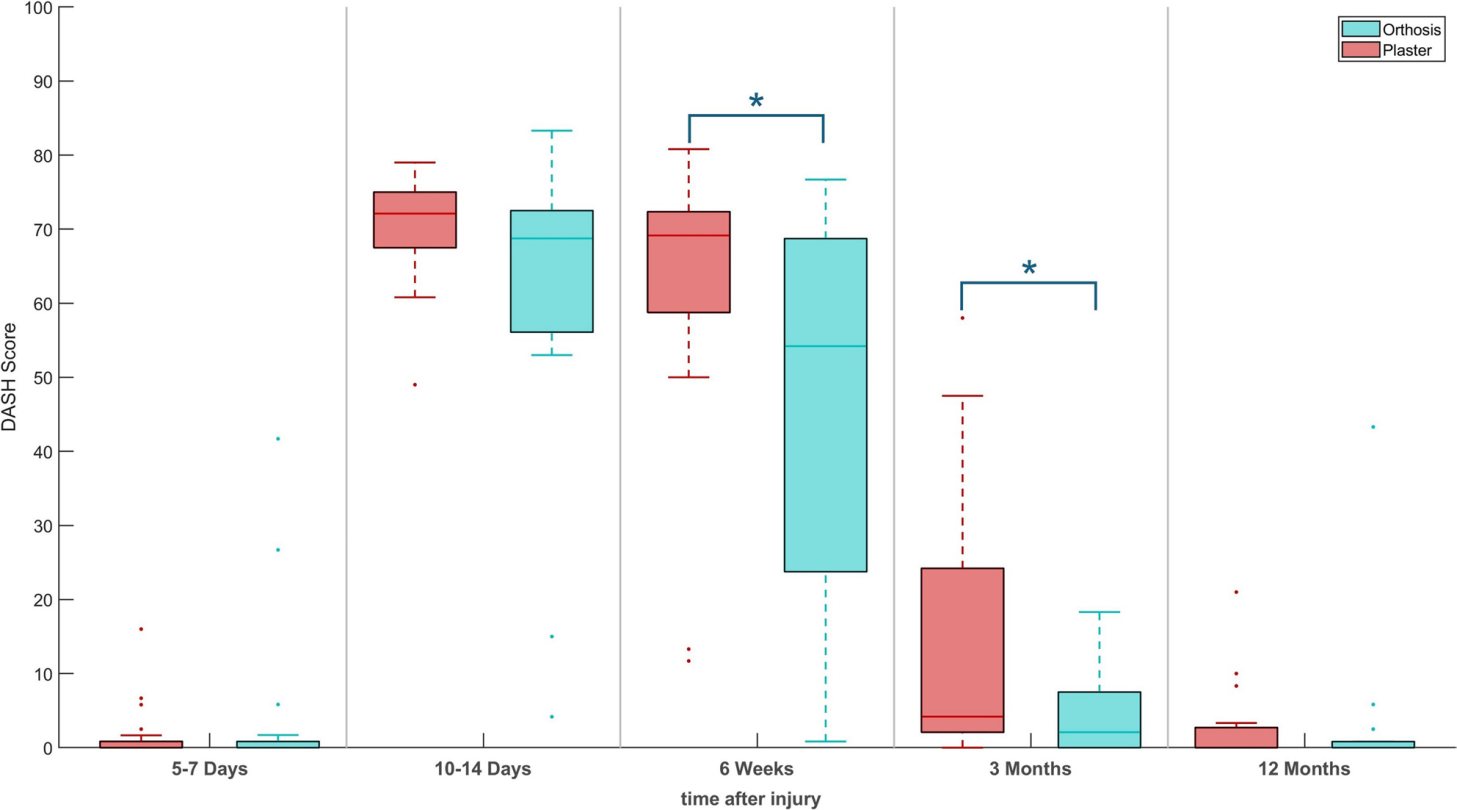
DASH score during patient rehabilitation. * denotes a sig. difference between plaster and orthosis treatment (*p*<0.05)

At the beginning of the study and after one year at the last follow-up examination, no significant difference between the two groups could be detected. 6 weeks (*p* = 0.034) and 3 months after injury (*p* = 0.024), the OG achieved significantly better results compared to the CG (Fig. 3). Mean results of DASH Score are presented in Table 3.

### Application time

**Fig. 4:**
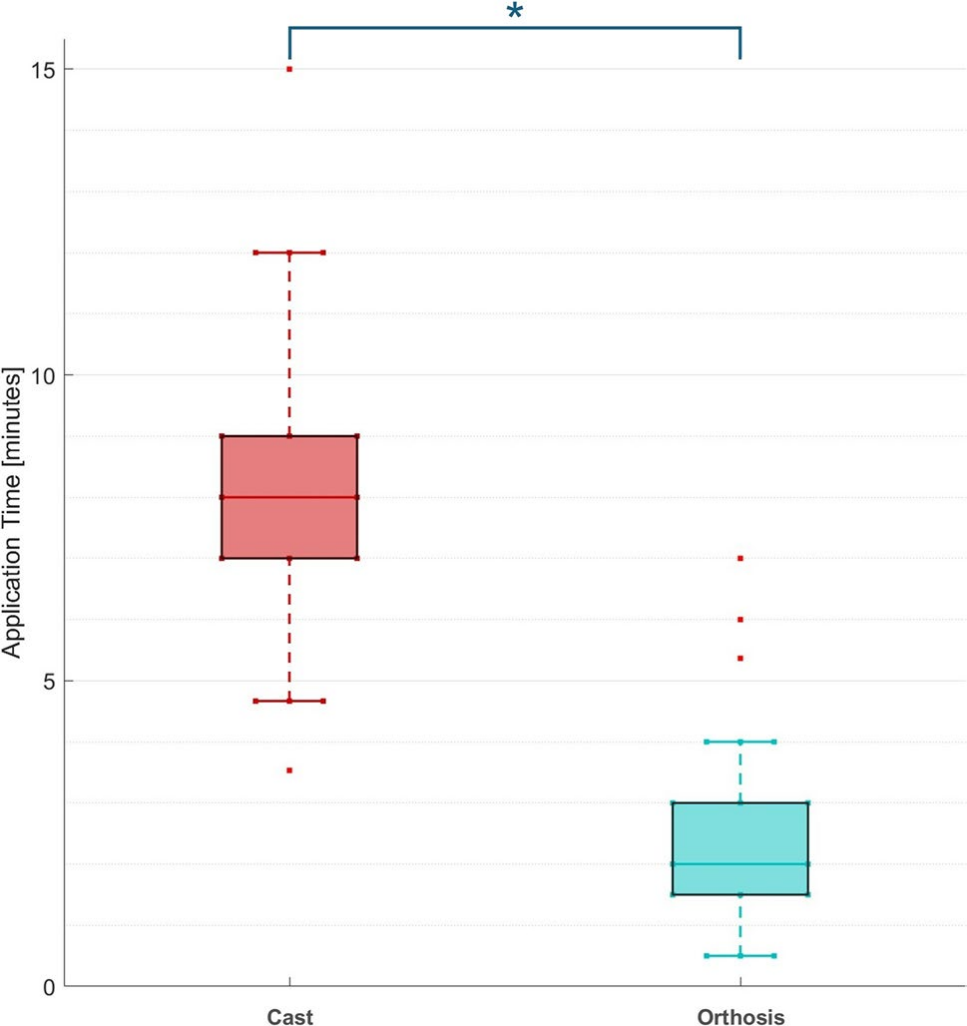
Application time of plaster (without time to harden) and orthosis. * denotes a sig. difference between plaster and orthosis treatment (*p*<0.05)

The application time was significantly shorter for the orthsis at 02:35 ± 1:53 min (*p* < 0.001) and almost three times faster than the application time of the cast (07:35 ± 2:25 min, time for hardening excluded; Fig. 4).

### Clinical function

Grip strength was measured from the sixth week of treatment onwards. A significant difference between both groups was measured 6 weeks and 12 months after injury with higher grip strength measured in the OG. For left-sided fractures, there was no significant difference between the OG and CG. Whereas right-sided fractures showed a significant difference after six weeks (*p* = 0.006) and after one year (*p* = 0.047) in favour of the orthosis. At 3 months follow-up, no significant difference between OG and CG (*p* > 0.05) could be observed in grip strength. 

From week 6 onwards and using the neutral-0-method, the range of motion (ROM) was measured as dorsal extension, palmar flexion, radial abduction, and ulnar abduction. For patients who had fractured their left wrist, there was no significant difference in dorsal extension (*p* > 0.385), palmar flexion (*p* > 0.085), radial abduction (*p* > 0.335), and ulnar abduction (*p* > 0.256). Similarly, for patients with a fracture of the right wrist, there was no significant difference in dorsal extension (*p* > 0.643), radial abduction (*p* > 0.868) and ulnar abduction (*p* > 0.714). However, the first ROM measurement showed a significant difference (*p* = 0.008) in palmar flexion in favour of the OG, while the palmar flexion did not significantly differ between both groups after 3 and 12 months (*p* > 0.067). Mean results of clinical function are presented in Table 3.

### Drop out

The present study included 12 drop out cases (22.6%) out of a total of 53 subjects. The drop out cases were distributed equally between both groups (6 orthoses vs. 6 casts) with no significant difference between the OG and CG (*p* = 0.941). The most common reasons for drop out were surgical indication after reduction, device application and CT imaging (CG: *n* = 2; OG: *n* = 3, *p* > 0.05) and secondary dislocation (see primary endpoint) during the first two weeks after injury (5–7 days post injury: n_CG_ = 1, n_OG_ = 2; 10–14 days post injury: n_CG_ = 1, n_OG_ = 1). Two subjects of the control group were excluded before the 3 months follow up due to moving abroad and a diagnosed known tumour disease (Fig. 1). No other adverse events were reported during study conduction.

## Discussion

DRF is one of the most common fractures in the Western world, with increasing incidence in older age groups [1, 26]. Especially in older patients, it can lead to functional limitations, loss of mobility, and reduced quality of life. Against this background, the search for effective, patient-friendly, and resource-saving treatment options is becoming increasingly important. Thus, the aim of the present study was to evaluate the clinical performance of the functional hand orthosis OPTIVOhand^®^ compared to conventional plaster casts in the conservative DRF treatment. The primary endpoint, maintenance of the reduction result, revealed no significant relationship between the type of immobilization and secondary dislocation. The numbers of secondary dislocations were comparable in both groups. Furthermore, the patients of the OG showed significantly better results in DASH score after 6 weeks and 3 months. Additionally, the OG reported significantly higher suitability for everyday use, hygiene, and wearing comfort.

A frequently discussed topic in treatment with orthoses is the potentially increased risk of secondary dislocations. For example, a study conducted by Janzig et al. using a 3D-printed orthoses had to be terminated prematurely due to a high rate of secondary dislocations (2 out of 5 patients) [9]. In this study, secondary dislocations occurred with approximately the same frequency in the OG and in the CG suggesting comparable retention stability. A3 and B2 fractures according to the AO classification were particularly affected by secondary dislocations. According to Lafontaine, these fracture types are associated with an increased risk of dislocation due to metaphyseal fracture zones. Furthermore, age over 60 years, a dorsal angulation over 20 degrees, intraarticular fractures and concomitant ulna fractures increase the risk of secondary dislocation [27]. Hence, in DRF of these classifications, the question about the right treatment is not “plaster cast versus orthosis” but rather “conservative vs. operative treatment”. Especially if three or more Lafontaine criteria are positive, surgical treatment is highly recommended. Careful indication therefore appears essential to use the orthosis safely in conservative treatment, primarily in stable fractures.

The study by Klopfer et al. focused on preoperative stabilization and the postoperative phase after palmar plate osteosynthesis comparing the OPTIVOhand^®^ orthosis to plaster. The results also showed comparable outcomes for both treatment methods in terms of pain progression, functionality, and patient satisfaction. This study examined the question of primary conservative therapy [23]. Thus, the present study expands the existing evidence on orthosis use and transfers the positive results of Klopfer et al. [23] to the conservative treatment concept for DRF.

Significantly higher patient satisfaction was observed in the OG. Patients in the OG reported fewer limitations in their everyday lives. In particular, the areas of hygiene, dressing, and adaptability were rated favourably. This assumption is supported by the subjective scores SF-36 and DASH, as these questionnaires specifically capture parameters relevant to everyday life. Further, the enhanced clinical function after end of immobilization may indicate a more functional treatment by the orthosis with less restrictions in daily living compared to cast immobilization. Next to the overall difference in grip strength between OG and CG 6 weeks after injury, there was a laterality effect observed with a significant group difference in right sided fractures but not in left-sided fractures. We assume that this difference results from the combination of right-handedness, which is mostly dominant in Germany, and less restrictions in daily use by the orthosis. Thus, right-handed patients were able to use the injured right hand more often despite of the orthosis compared to the cast. While right-handed patients with left-sided DRF had a lower usage in activities of daily living (e.g. still brushing teeth with the non-injured, dominant hand).

Unlike traditional plaster casts, which immobilize the wrist in a circular and rigid position, modern orthoses enable functional immobilization with greater flexibility thanks to strategically placed pressure points and adjustable splint elements, which promote comfort and functional preservation. At the same time, it reduces the risk of pain or pressure points associated with wearing a cast. This benefits both patients and medical staff. The significantly reduced application time and the high adaptability of the orthosis facilitate workflows in everyday clinical practice [23]. Another advantage is that orthoses can also be applied by less experienced staff after appropriate manufacturer training, which can lead to a noticeable reduction in the workload of medical staff when there is a high volume of patients in the emergency room. Despite the promising results reported in the literature, orthoses are not yet part of standard treatment.

When interpreting the findings, the limitations of the study should be acknowledged. At first, the imbalance of age distribution in both groups might have an influence especially on secondary endpoints. Hereby, a higher age is often associated with worse subjective and objective functionality. However, the short and mid-term effects might still be related to the type of immobilization as initial scores and long-term results reveal no significant differences between both groups. It should be noted that the imbalance was caused by chance as a result of the randomization process. Further, with the visible type of treatment, the blinding of patients in the CG was not possible. With limited time resources in the trauma centre, the effort to blind the medical staff was not feasible during clinical practice. Thus, the missing blinding might lead to potential bias on both sides. On one side, patients of the CG could have been disappointed not receiving the desired orthosis and therefore gave a poorer subjective assessment in the patient reported outcome measures. On the other side, the medical staff involved might be biased by a potential conflict of interest, favouring patients with orthosis. To minimise this conflict of interest, the funding was granted to the institution and not the medical staff involved. The manufacturer´s influence on study preparation, conduction, analysis and interpretation was ruled out. Last, the relatively small sample size and the high number of drop out cases reduced the statistical power of this study because of rigorous inclusion and exclusion criteria and the per-protocol analysis of only complete cases. Patients were excluded if surgery was indicated after reduction, immobilization and CT-scan; this occurred more often than expected (*n* = 5). Due to the equal distribution of drop out cases there was no significant correlation between drop out and treatment group (*p* = 0.941). The calculated effect size according to Cramer’s V was V = 0.010, which represents a negligible effect. Hence, a distortion of the study results due to drop out is not to be assumed.

## Conclusions

This clinical randomized controlled study compared the immobilization using orthosis (OPTIVOhand^®^) with traditional plaster cast for conservative DRF treatment. Both treatment methods were comparable in terms of maintenance of reduction result and pain progression. However, the orthosis showed significantly better results in terms of functionality, hygiene, application time, and patient satisfaction. Complications such as secondary dislocations occurred at almost the same frequency in both groups. With respect to the monocentric design, the small sample size and the limitations listed, the results highlight the potential of orthoses as a good alternative to conventional treatment with plaster casts.

## Supplementary Information

Supplementary Material 1.

Supplementary Material 2.

## Data Availability

A minimal data set of the current study can be requested from the corresponding author.
