# Supplementary material for: Outcome analysis of conservative treatment of a distal radius fracture with OPTIVOhand orthosis versus plaster cast: a randomized controlled trial
Source: BMC Musculoskelet Disord. 2026 Feb 11;27:172. doi: 10.1186/s12891-026-09585-4 (PMC12930803; doi:10.1186/s12891-026-09585-4)
Supplement: Supplementary file 2 — Supplementary Material 2. [file 12891_2026_9585_MOESM2_ESM.pdf]

Study-specific questionnaire

General physical condition

	(5) = does not apply at all	(4) = does not apply	(3) = applies to a limited extent	(2) = applies	(1) = absolutely true
Has the pain in your wrist decreased since your last appointment?	☐	☐	☐	☐	☐
Do you feel limited in the performance/functionality of your wrist?	☐	☐	☐	☐	☐
Do you feel limited in your overall physical capacity?	☐	☐	☐	☐	☐

Functionality orthosis/splint

	(5) = does not apply at all	(4) = does not apply	(3) = applies to a limited extent	(2) = applies	(1) = absolutely true
Was the orthosis/splint easy to use?	☐	☐	☐	☐	☐
Did the orthosis/splint make you feel restricted in terms of personal hygiene?	☐	☐	☐	☐	☐
I felt that the orthosis/splint fit my wrist very well?	☐	☐	☐	☐	☐
I had the feeling that the orthosis/splint was causing pressure discomfort.	☐	☐	☐	☐	☐
Does the orthosis/splint bother me in terms of appearance (aesthetics)?	☐	☐	☐	☐	☐
Did I have restrictions on what I could wear?	☐	☐	☐	☐	☐
